# Spatiotemporal optical vortices with arbitrary orbital angular momentum orientation by astigmatic mode converters

**DOI:** 10.1515/nanoph-2021-0496

**Published:** 2022-02-16

**Authors:** Yimin Zang, Amal Mirando, Andy Chong

**Affiliations:** Department of Electro-Optics and Photonics, University of Dayton, Dayton, USA; Department of Physics, University of Dayton, Dayton, USA

**Keywords:** arbitrary OAM, cylindrical lens system, STOVs

## Abstract

We generate a spatiotemporal optical vortex (STOV) with tunable orbital angular momentum (OAM) orientation by a simple lens system. We utilize a cylindrical lens system, which is an astigmatic mode converter, to add longitudinal angular momentum to tilt the purely transverse OAM in an arbitrary direction. The amount of tilt is tunable by adjusting the lens system, and thus the OAM direction is continuously adjustable. STOVs with adjustable OAM directions have been verified theoretically and experimentally. We believe such direction controllable OAMs will enrich future applications.

## 1 Introduction

Structured light has various applications due to its degree of freedom in spatial and spatiotemporal structure [1]. As a type of structured light, optical vortices have a wide range of applications [2], such as super-resolution imaging [3], optical communication [4], quantum information [5, 6], optical tweezers, trappers, and spanners [7–9], etc. Such spatial optical vortex beams carry longitudinal orbital angular momentum (OAM) [10]. Recently, novel optical vortices, so-called spatiotemporal optical vortices (STOVs) with the OAM transverse to the propagation direction have been demonstrated [11, 12]. Since demonstrated optical vortices can possess longitudinal or transverse OAM, a natural next step is to pursue a wave packet which carries OAMs in arbitrary directions. STOVs with OAM in arbitrary directions may have unique applications. For example, such STOVs can be useful in communication to enhance the data rate by multiplexing the OAM orientations as independent data states [13, 14]. This tilted OAM can be interesting in nonlinear applications such as the second harmonic generation for the arbitrary direction OAM conservation [15, 16]. The spontaneous parametric down-conversion may be able to generate entangled tilted OAM states [17, 18]. As such a vortex has a tunable OAM orientation, it can be useful for unique light–matter interactions. For example, the tilted OAM can sculpt the unique photocurrent distribution in a semiconductor with more degrees of freedom by transferring arbitrary OAM direction [19, 20].

Today, the investigation of arbitrary STOV OAM direction is ongoing. The STOV with a unique OAM direction has been proposed in a theoretical study by the relativistic Hall effect [21]. Recently, the direct combination of the longitudinal and transverse OAM in an optical wave packet by using two spatial light modulators (SLMs) has been proposed and demonstrated [22]. Such wave packet has two perpendicularly crossed vortex lines resulting in a controllable magnitude and direction of OAM. Recently, a photonic crystal slab structure to generate STOVs with arbitrary OAM direction has been proposed theoretically [23].

Here, we propose a simple method to generate a STOV with a continuously tunable OAM direction, instead of using multiple SLMs or designing a photonic crystal structure, utilizing a cylindrical lens system to provide a longitudinal torque and manipulate OAM direction is more flexible and cost-efficient. We demonstrate that the OAM direction can be fine-tuned by adjusting the lens system. The additional longitudinal torque couples to the original transverse OAM to adjust the OAM direction. The experimental results agree very well with the theoretical predictions. We strongly believe that this work provides unique optical vortices for future applications prior mentioned.

## 2 Theoretical analysis

Researchers have demonstrated utilizing the cylindrical lens pair as an astigmatic mode converter to realize the conversion between transverse modes, particularly Hermite–Gaussian and Laguerre–Gaussian modes [24]. Theoretical work also proves that mode converters can provide longitudinal torque to the incoming beams under this process [24, 25]. Inspired by this mode converter, it is worthy to investigate the effect of a cylindrical lens pair on a STOV with a purely transverse OAM. The scalar field of STOV with the spiral phase on the spatiotemporal (*y* − *t*) domain is expressed in Eq. (1):

(1) $$E\left(x,y,t\right)={\left(y\pm it\right)}^{\left|l\right|}{\text{e}}^{-x^{2}/{w_{1}}^{2}-y^{2}/{w_{2}}^{2}-t^{2}/{w_{3}}^{2}}$$

**Figure 1:**
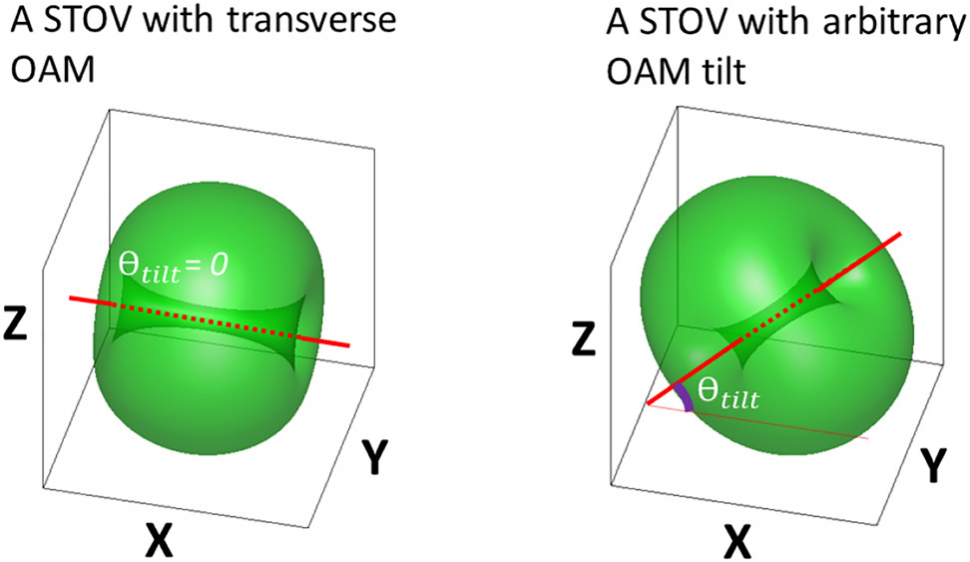
Conceptual sketch to show the tilt angle *θ*_tilt_, left: a STOV with transverse OAM, *θ*_tilt_ = 0; right: a STOV with OAM tilt, *θ*_tilt_ can be some arbitrary value, *x*–*y* is the transverse plane.

The integer *l* represents the topological charge while the three-dimensional (3D) widths of the wave packet are defined by *w*_1_, *w*_2_, and *w*_3_. Such a STOV carries a purely transverse OAM in $\vec{x}$ direction. In contrast, one can envision a STOV with the OAM direction ‘tilted’ from the transverse direction as shown in Figure 1. We define the amount of tilt as the angle of the OAM direction with respect to the transverse direction, which is denoted by *θ*_tilt_ in Figure 1.

**Figure 2:**
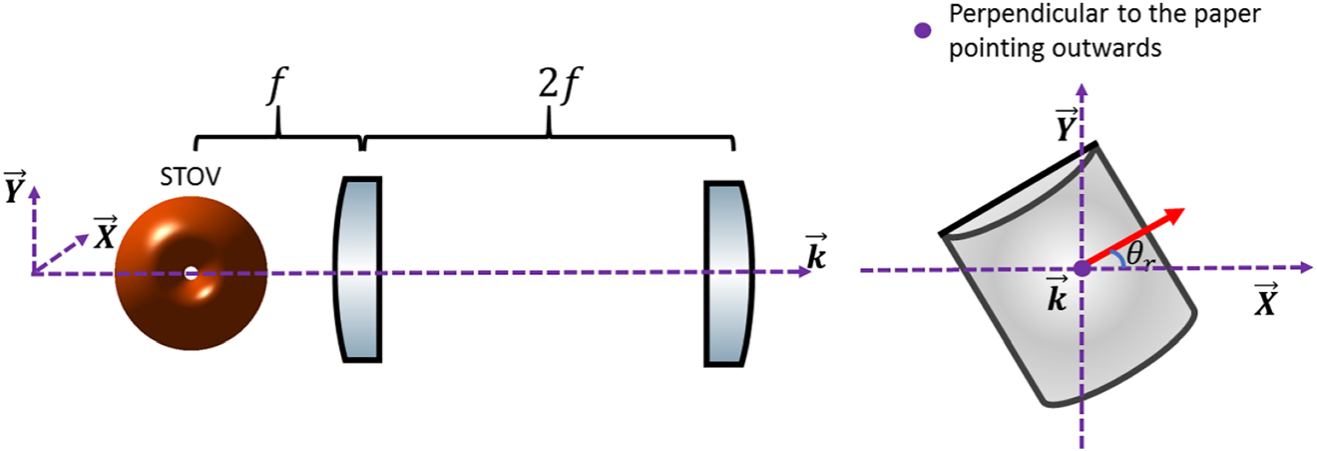
Sketch of a STOV going through a rotational cylindrical lens pair; axis of lens has a rotational angle *θ*_r_ with respect to positive $\vec{x}$ axis.

Figure 2(a) shows a schematic of the STOV propagating through a cylindrical lens mode converter. The horizontal axis of the cylindrical lens is marked by a red arrow as in Figure 2(b). The angle between the lens axis and the *x*-axis is defined as the rotational angle *θ*_r_ as in Figure 2(b). For the incoming STOV, the phase provided by a cylindrical lens can be expressed as Eq. (2):

(2) $$\Phi \left(x,y\right)=P\left(x,y\right){\text{e}}^{-\text{i}\frac{k}{2f}{\left(x\;\cos\;\theta_{\text{r}}-y\;\sin\;\theta_{\text{r}}\right)}^{2}}$$

where *P*(*x*, *y*) is the pupil function, and we can set *P*(*x*, *y*) = 1 because the beam size is much smaller than the diameter of the lens.

One can calculate the OAM change with an analogy between paraxial optics and quantum mechanics [25]. Equation (3) shows the additional longitudinal OAM operator $\delta {\hat{L}}_{z}\left(\theta_{\text{r}}\right)$ by a cylindrical lens with a rotational angle *θ*_r_.

(3) $$\delta {\hat{L}}_{z}\left(\theta_{\text{r}}\right)=\frac{k}{2f}\left[2\hat{x}\hat{y}\;\cos\;2\theta_{\text{r}}+\left({\hat{x}}^{2}-{\hat{y}}^{2}\right)\;\sin\;2\theta_{\text{r}}\right]$$

where $\hat{x}$ and $\hat{y}$ are position operators. The longitudinal OAM change for a transverse cross-section (at a certain time) of the STOV (*l* = 1) is expressed in (4):

(4) $$\begin{array}{ll} \left\langle E|\delta {\hat{L}}_{z}\left(t\right)|E\right\rangle & \propto \iint \left(E^{*}\delta {\hat{L}}_{z}E\right)\text{d}x\text{d}y\\ & =\frac{\pi w_{1}w_{2}k}{64f}{\text{e}}^{-\frac{2t^{2}}{w_{3}^{2}}}\left[3w_{1}^{2}+4t^{2}\frac{w_{1}^{2}}{w_{3}^{2}}\right.\\ & \left.\;-w_{2}^{2}-4t^{2}\frac{w_{2}^{2}}{w_{3}^{2}}\right]\;\sin\left(2\theta_{\text{r}}\right) \end{array}$$

To obtain the total longitudinal OAM change, Δ*L*_*z*_, we let *w*_1_ = *w*_2_ = *w* and integrate cross-sectional OAMs in time. The Δ*L*_*z*_ of the whole wave packet by a cylindrical lens is proportional to the sine of the 2*θ*_r_ as in Eq. (5).

(5) $$\Delta L_{z}=\int \left\langle E|\delta {\hat{L}}_{z}\left(t\right)|E\right\rangle dt\propto \frac{kw^{4}w_{3}}{f}\sin\left(2\theta_{\text{r}}\right)$$

Apparently, the longitudinal OAM change is adjustable by the controlling cylindrical lens orientation. One can extend the calculation for the whole cylindrical lens pair to obtain the longitudinal OAM change [24, 25]. However, the analytic expression for the lens pair system turns out to be quite complicated. Instead, we rely on numerical simulations to evaluate the longitudinal OAM changes for the whole lens system. Numerically calculated OAM of the entire wave packet is the integral of the OAM density, which can be written as Eq. (6) [10]:

(6) $$L_{\text{total}}=∭r\times g\;d^{3}r$$

where **r** is the position vector and the linear momentum density **g** of the wave packet can be calculated according to the phase gradient Eq. (7) [10]:

(7) $$g=i\omega \frac{\epsilon_{0}}{2}\left(u\nabla u^{*}-u^{*}\nabla u\right)+\omega \epsilon_{0}k|u{|}^{2}z$$

where *u* is the complex scalar amplitude of the wave packet and *k* is the propagation constant. Starting from a standard STOV shown in Eq. (1), we can numerically simulate the STOV after the lens system to evaluate the direction and the magnitude of the OAM.

**Figure 3:**
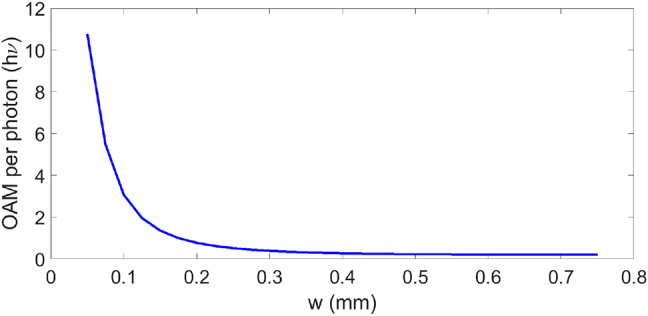
Average longitudinal OAM per photon gained by a 4*f* cylindrical lens pair for input STOVs with different spatial radius.

To match experimental parameters, we choose the pulse width *w*_3_ = 0.4 ps. The beam size is critical to induce a large longitudinal OAM change. Figure 3 shows the average longitudinal OAM change per photon induced by the two-cylindrical lens system versus the beam width *w*. It is clear that the smaller beam size is preferred to increase the longitudinal OAM change. The curve indicates that the input beam size *w* = 0.7 mm on the 4*f*-cylindrical lens system does not have much longitudinal OAM effect. Thus, an additional spherical lens is applied to reduce the input beam size to *w* = ∼0.14 mm (beam radius in *x*-direction based on the half-width of 1/*e*^2^ of the peak intensity) when it reaches the first cylindrical lens of the 4*f*-cylindrical lens pair. The result is improved to induce a longitudinal OAM/photon of 1.7*hν*.

Even though a single cylindrical lens can generate the tiled STOV, there are some disadvantages of a single lens setup. The simulation study indicates that a large tilt angle is only observable within a short-range near the focal point. The small beam size at the focus makes the measurement quite challenging at the same time. Therefore, we used a spherical lens with a 4*f* cylindrical lens pair which generates a STOV with a larger beam size while the large tilted angle is observable for a longer range.

## 3 Generation of STOVs with arbitrary OAM tilt

### 3.1 Experimental setup

**Figure 4:**
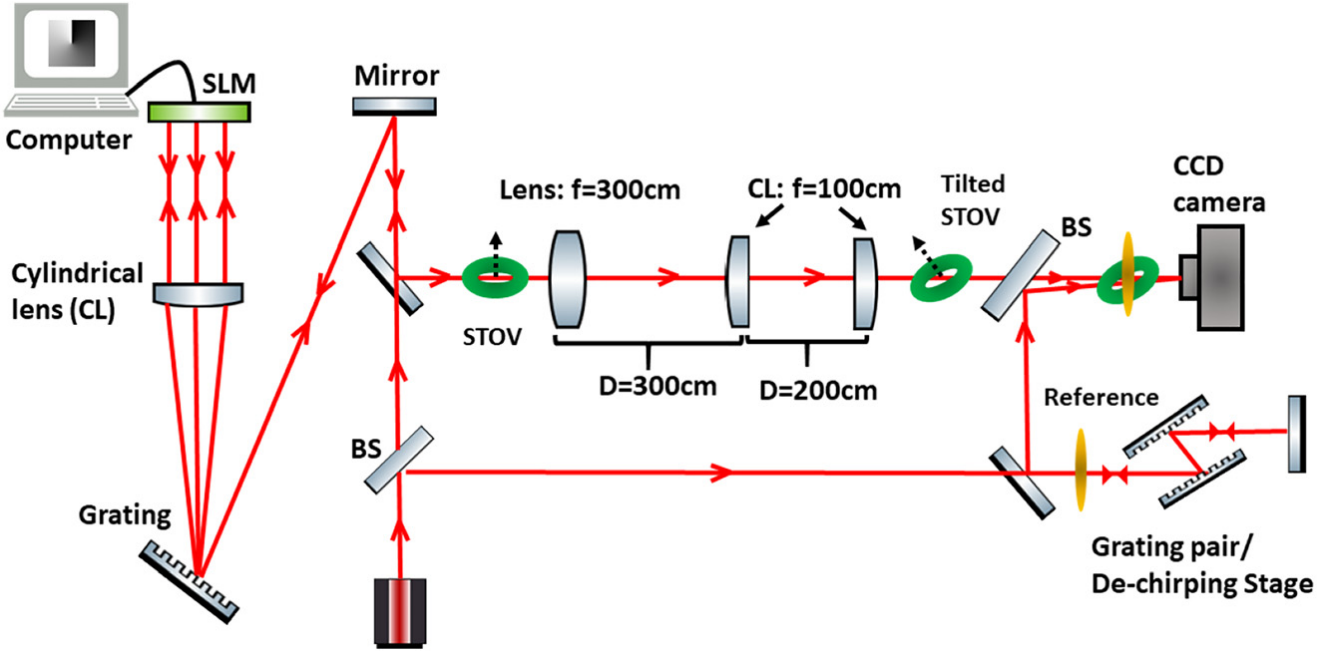
Sketch of the experimental setup for generating and detecting STOVs with arbitrary OAM tilt, BS: beam splitter.

The schematic of the experimental setup is shown in Figure 4. The process of generating and diagnosing the STOV with an arbitrary OAM tilt contains three parts: first, we generate a STOV with a purely transverse OAM by following the procedure demonstrated in [11]. In the experiment, a mode-locked fiber laser provides femtosecond pulses for the pulse shaper. A spiral phase is applied to the SLM in the pulse shaper to form STOVs [11]. Second, a three-lens system, which is the combination of a converging lens and a cylindrical lens pair, is used to convert the original STOV into the tilted STOV. In the three-lens system, a regular lens with a focal length *f*_1_ = 300 mm converges the incoming STOV on the first cylindrical lens with *f*_2_ = 100 mm. The second cylindrical lens, which is identical to the first one, is separated from the first lens by a distance of 2*f* = 200 mm. Two cylindrical lenses have the same rotational angle denoted by *θ*_r_ which is the main parameter that controls the OAM orientation. Finally, by applying 3D measurement technology [26], we can reconstruct the iso-intensity profile of the wave packet. Since the STOV after the lens system is no longer collimated, we measured the wave packet profile at the back focal plane of the lens system, where the spatial size is at its minimum.

### 3.2 Simulation and experimental results

**Figure 5:**
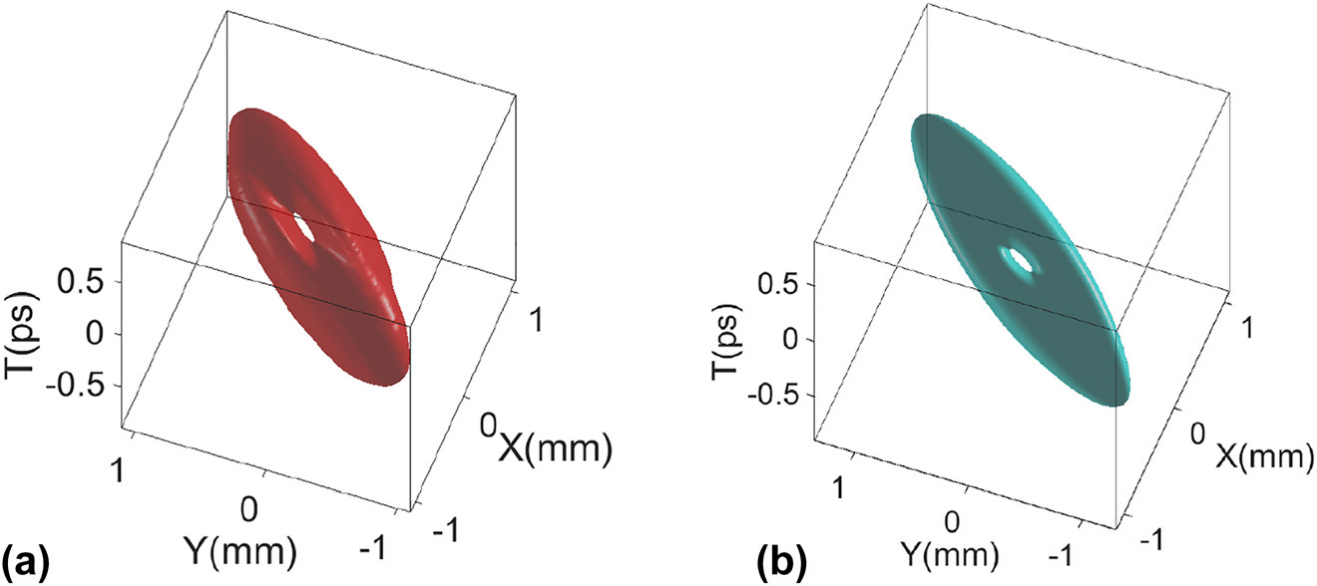
Experimental and simulated iso-intensity plot of the STOV with OAM tilt generated by cylindrical lens system with rotational angle *θ*_r_ = 45°: (a) experimental result; (b) simulation result.

We experimentally generate a tilted STOV with *l* = 1 first. To clearly present the timescale of the STOV, we present iso-intensity figures in the *x* − *y* − *t* coordinate system. Figure 5 shows a typical STOV measured with a tilted OAM when the rotational angle of the lens system is *θ*_r_ = 45°. We can observe that the OAM is clearly tilted from the purely transverse direction.

**Figure 6:**
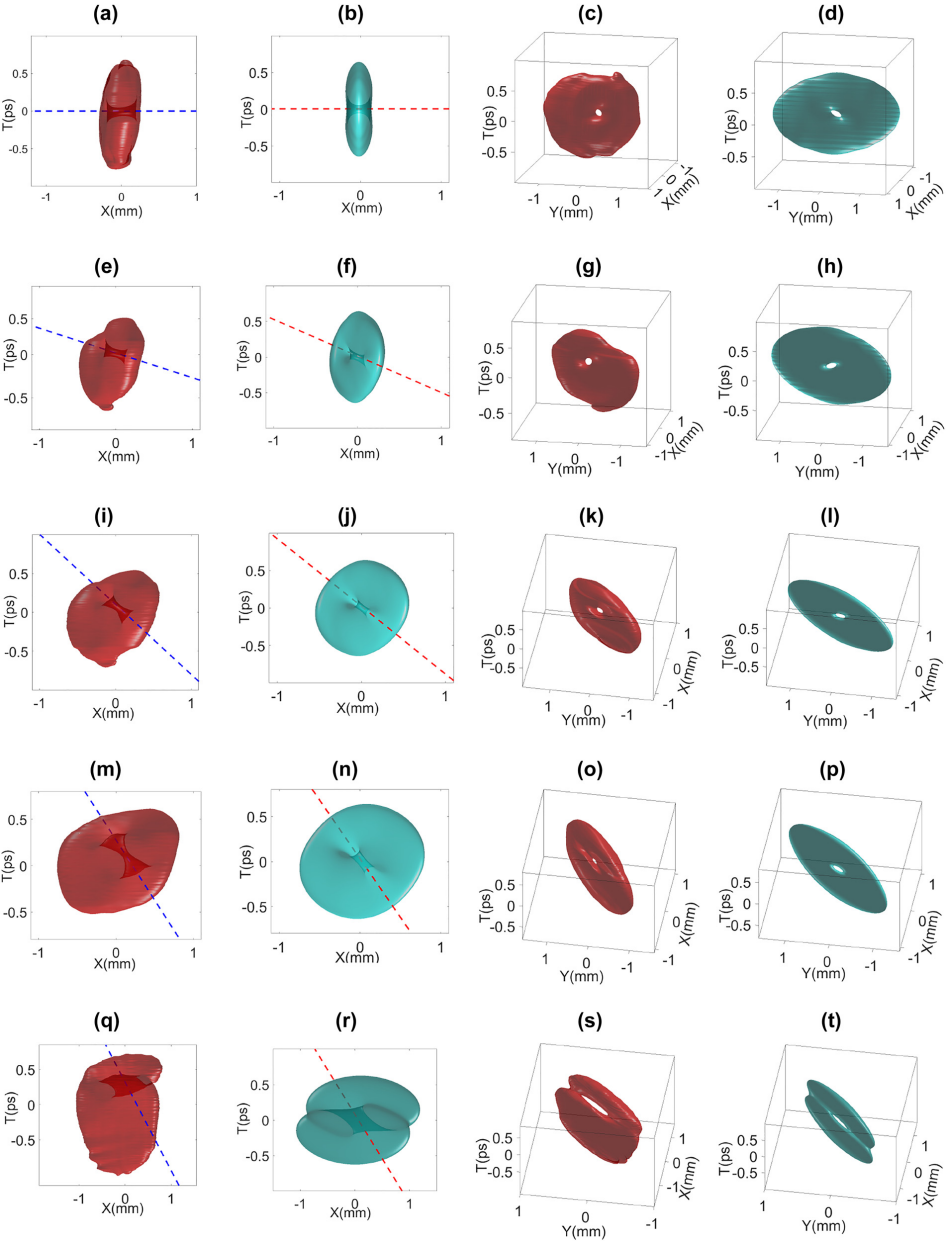
Experimental and simulated iso-intensity plots of the STOV with arbitrary OAM tilt generated by cylindrical lens system with different rotational angles. STOVs in red represent experimental data and STOVs in green represent simulation results. Two views of iso-intensity plots are demonstrated: *x* − *t* domain view and the three-dimensional view (viewpoint is adjusted according to the tilt of the STOV: (a)–(d): *θ*_r_ = 0; (e)–(h): *θ*_r_ = 10°; (i)–(l): *θ*_r_ = 20°; (m)–(p): *θ*_r_ = 45°; (q)–(t): *θ*_r_ = 60°; Dashed line in color represents vortex line.

By rotating the cylindrical lens pair, we control the OAM tilt angle. The experimental and the simulations results are shown in Figure 6. We use dashed lines to highlight each phase singularity which represents the direction of the vortex line. From the *x* − *t* domain view in Figure 6, we can clearly recognize the changing of the vortex line direction, which indicates the local OAM orientation. Starting from the vortex line along $\vec{x}$ axis, as *θ*_r_ increases, we can observe the vortex line moving towards the longitudinal direction ( $\vec{t}$ axis) which can be interpreted as the addition of the longitudinal OAM by the lens system. We also provide the three-dimensional view to demonstrate the overall donut shape structure of tilted STOVs. As *θ*_r_ > 60°, the wave packet shows a strong tendency to split into two lobes. Such a phenomenon can be interpreted by the lensing effect. The spiral phase of the input STOV is in the *y* − *t* domain and as the cylindrical lens rotation angle gets close to 90° to have a strong focus in *y*-direction, the wave packet has a tendency to split into two-lobes due to strong spatiotemporal astigmatic focusing effect [27]. For rotational angles *θ*_r_ > 90° cases, the behavior of the tilted STOV is symmetric to 0°–90° due to the symmetry of the lens system.

**Figure 7:**
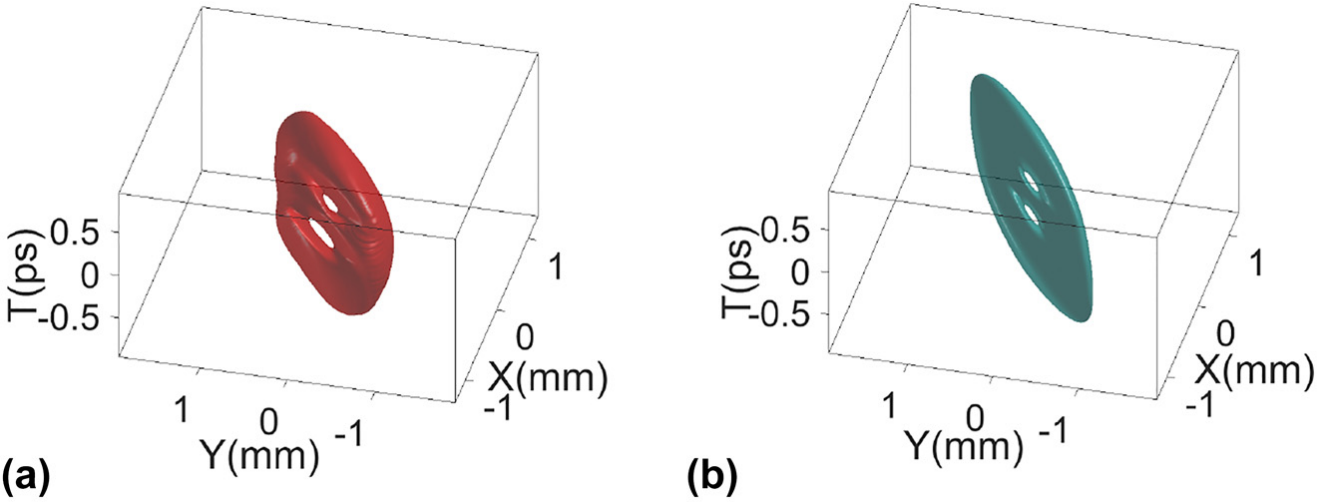
Three-dimensional view of iso-intensity plots of the STOV with OAM tilt for topological charges *l* = 2, *θ*_r_ = 45°: (a) experimental result; (b) simulation result.

Figure 7 shows the tilted STOV with topological charge *l* = 2. Two vortex holes appear at its center due to spatiotemporal astigmatism [11]. Experimental and simulation iso-intensity plots of the tilted STOV with topological charge *l* = 2, generated by the three lens system with cylindrical lens rotation of 45° are shown in Figure 7(a) and (b).

### 3.3 Results analysis

**Figure 8:**
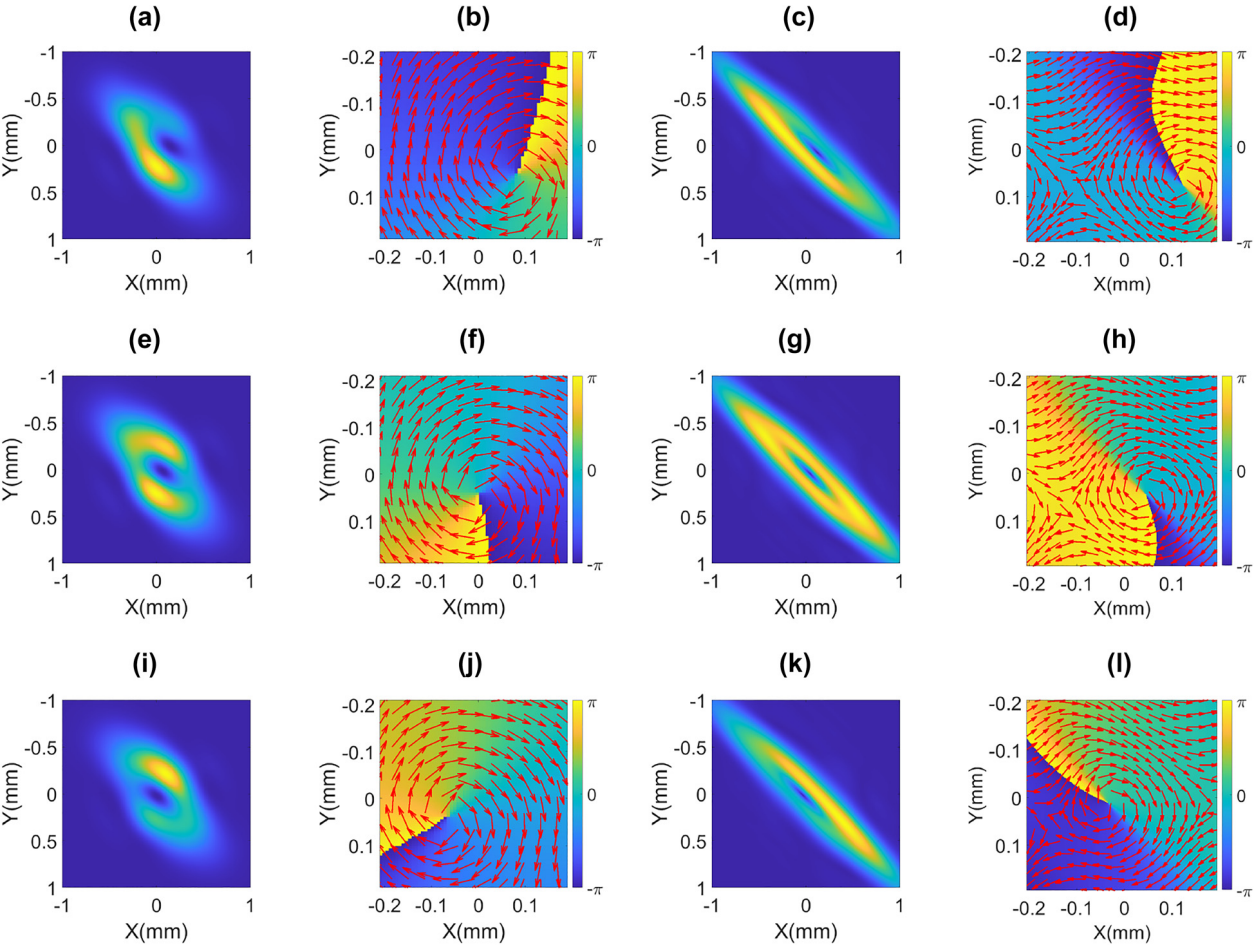
Simulation and experimental results of intensity and unit vector to show Poynting vector direction of transverse planes at different temporal (longitudinal) positions: (a) experimental intensity at *t* = −66.7 fs; (b) experimental phase and vector plot at *t* = −66.7 fs; (c) simulation intensity at *t* = −66.7 fs; (d) simulation phase and vector plot at *t* = −66.7 fs; (e) experimental intensity at *t* = 0 fs; (f) experimental phase and vector plot at *t* = 0 fs; (g) simulation intensity at *t* = 0 fs; (h) simulation phase and vector plot at *t* = 0 fs; (i) experimental intensity at *t* = 66.7 fs; (j) experimental phase and vector plot at *t* = 66.7 fs; (k) simulation intensity at *t* = 66.7 fs; (l) simulation phase and vector plot at *t* = 66.7 fs.

For further analysis, we take the tilted STOV generated at *θ*_r_ = 45° and examine the local momentum vector field on the transverse profile at different times. According to Eq. (7), the linear momentum density has two terms: the second term *ωϵ*_0_*k*|*u*|^2^ ***z*** represents the linear momentum of the wave packet along the propagation direction, while the first term $i\omega \frac{\epsilon_{0}}{2}\left(u\nabla u^{*}-u^{*}\nabla u\right)$ is the relative momentum density distribution on the spatiotemporal domain as shown in Figure 8. By scanning a short reference pulse at different temporal locations of the STOV, we can measure the spatial intensity and the phase at different temporal locations of the STOV. The experimental and the simulation results at various temporal locations are shown in Figure 8. Figure 8 shows a good agreement between the experimental measurement and numerical simulations. We can see a clear donut structure, where the momentum density circulates. Within the temporal range of 0.133 ps, the singularity location moves across the beam center in *x*-direction with an amount of ∼0.155 mm, which is consistent with the tilted vortex shown in Figure 6(n). This is a distinct feature of the tilted STOV.

**Figure 9:**
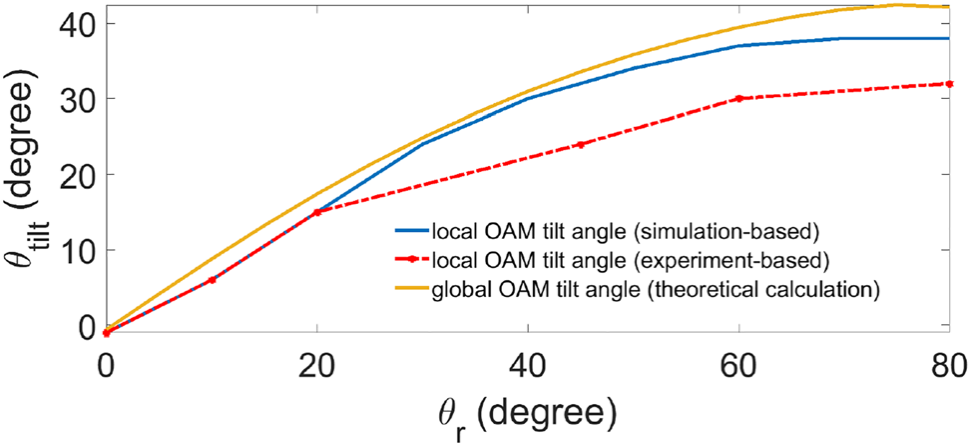
Simulation and experimental data of tilted angle evolution as the function of rotational angle. The tilt angle is calculated based on the OAM direction, where the local OAM direction is determined by the hole singularity direction at the center, both by simulation (blue line) and experiment (red-dashed line); the global OAM is based on simulation calculated OAM of the overall wave packet (yellow line).

The vortex line direction in the iso-intensity plots indicates the local OAM direction. To calculate the tilt angle *θ*_tilt_, we used 3D space coordinate *x* − *y* − *z* where *z* = −*ct* and aligned a reference vector along the singularity. This direction of the vortex line is the local OAM direction. Figure 9 shows the simulation and experimental results of *θ*_tilt_ as a function of the lens rotational angle *θ*_r_. From these results, we can see the local OAM tilt angle can reach 30° which is well verified by the experiment. Besides the local OAM, the numerically calculated total OAM of the entire wave packet based on Eq. (6) is a three-dimensional vector (strictly speaking, pseudovector), which also has a tilt angle *θ*_tilt_ with respect to the transverse plane. Generally speaking, the tilt angle determined by local and total OAM is different, as the wave packet has an astigmatic phase induced by the lens system with longitudinal OAM components not related to the local OAM near the singularity [28]. Both local and total OAM results indicate that we can conveniently control the OAM direction by simply tuning the rotation of the cylindrical lens pair system.

## 4 Conclusions

We generate tilted STOVs with a continuously tunable OAM direction by controlling the rotational angle of the cylindrical lens pair which is an astigmatic mode converter. The tilted STOVs contain a mixture of longitudinal and transverse OAM which results in a tilted vortex line in a three-dimensional fashion. As a novel structured light, we strongly believe that the adjustable OAM direction will be useful in future applications. For example, it can be utilized in hybrid quantum entanglement as a photon source with flexible spatiotemporal structures [29]. In addition, there is potential to develop a compact, chip-scale device to generate the tilted STOVs. Such an integrated approach has practical significance in optical communication [30].
